# Diagnostic value of serum cathepsin S in type 2 diabetic kidney disease

**DOI:** 10.3389/fendo.2023.1180338

**Published:** 2023-05-25

**Authors:** Xuejing Ren, Wanqing Wang, Huixia Cao, Fengmin Shao

**Affiliations:** ^1^ Henan Key Laboratory of Kidney Disease and Immunology, Henan Provincial Clinical Research Center for Kidney Disease, Zhengzhou University People’s Hospital, Henan Provincial People’s Hospital, Zhengzhou, Henan, China; ^2^ Academy of Medical Sciences, Zhengzhou, Henan, China; ^3^ Health Management Centre, People’s Hospital of Zhengzhou University, Central China Fuwai Hospital, Zhengzhou, Henan, China

**Keywords:** type 2 diabetes mellitus, diabetic kidney disease, cathepsin S, risk factor, diagnostic value

## Abstract

**Background:**

Identification of risk factors that have causal effects on the occurrence of diabetic kidney disease (DKD), is of great significance in early screening and intervening for DKD, and in delaying the progression of DKD to end-stage renal disease. Cathepsin S (Cat-S), a novel non-invasive diagnostic marker, mediates vascular endothelial dysfunction. The diagnostic value of Cat-S for DKD has rarely been reported in clinical studies.

**Objective:**

To analyze whether Cat-S is a risk factor for DKD and evaluate the diagnostic value of serum Cat-S for DKD.

**Methods:**

Forty-three healthy subjects and 200 type 2 diabetes mellitus (T2DM) patients were enrolled. T2DM patients were divided into subgroups according to various criteria. Enzyme-linked immunosorbent assay was used to detect serum Cat-S levels among different subgroups. Spearman correlation analysis was used to analyze correlations between serum Cat-S and clinical indicators. Multivariate logistic regression analysis was performed to analyze risk factors for the occurrence of DKD and decreased renal function in T2DM patients.

**Results:**

Spearman analysis showed that serum Cat-S level was positively correlated with urine albumin creatinine ratio (r=0.76, *P*<0.05) and negatively correlated with estimated glomerular filtration rate (r=−0.54, *P*<0.01). Logistic regression analysis showed that increased serum Cat-S and cystatin C(CysC) were independent risk factors for DKD and decreased renal function in T2DM patients (*P*<0.05). The area under the receiver operating characteristic (ROC) curve was 0.900 of serum Cat-S for diagnosing DKD, and when the best cut-off value was 827.42 pg/mL the sensitivity and specificity were 71.6% and 98.8%, respectively. Thus, serum Cat-S was better than CysC for diagnosing DKD (for CysC, the area under the ROC curve was 0.791, and when the cut-off value was 1.16 mg/L the sensitivity and specificity of CysC were 47.4% and 98.8%, respectively).

**Conclusion:**

Increased serum Cat-S were associated with the progression of albuminuria and decreased renal function in T2DM patients. The diagnostic value of serum Cat-S was better than that of CysC for DKD. Monitoring of serum Cat-S levels could be helpful for early screening DKD and assessing the severity of DKD and could provide a new strategy for diagnosing DKD.

## 1 Introduction

Diabetic kidney disease (DKD) is one of the most common and serious microvascular complications of diabetes, with the incidence as high as 20%-40% in patients with type 2 diabetes mellitus (T2DM) (1). There are usually no obvious symptoms and signs in the early stage of DKD, which leads to delayed treatment, losing the best opportunity for early intervention and progressing to end-stage renal disease (ESRD) gradually. Therefore, the identification of risk factors with causal effects on the incidence of DKD is crucial for the prevention of the onset of DKD and also lays a foundation for achieving the early screening of DKD, reducing the morbidity rate and delaying progression of DKD to ESRD.

Renal biopsy pathology is the gold standard for the diagnosis of DKD, but it is not suitable for diagnosing early DKD because of the risk of bleeding. Therefore, non-invasive screening for diagnosing early DKD is more valuable for clinical studies. Some studies have suggested serum cystatin C (CysC) as an indicator for diagnosing early DKD, but this is controversial (2–4). Therefore, it is of great importance to explore novel non-invasive biomarkers for early diagnosing DKD.

Cathepsin S (Cat-S) was a secreted cysteine proteolytic enzyme that is mainly expressed in macrophages (5). Macrophages undergoing chemotaxis adhere to the basement membrane of blood vessels and secrete Cat-S, and the secreted Cat-S was involved in hydrolysis of elastin, laminin, collagen and other extracellular matrix components, causing vascular damage (6). The deficiency of Cat-S gene or the activity of Cat-S was inhibited were strongly associated with neovascularization (7). In recent years, studies have shown that upregulation of Cat-S was associated with the development of IgA nephropathy, lupus nephritis, insulin resistance, diabetes and other renal diseases (8–10). Monocyte/macrophage-derived Cat-S has been found to activate protease-activated receptor-2 on glomerular endothelial cells, causing endothelial damage, albumin leakage, inflammation and glomerulosclerosis (11). Inhibition of Cat-S has been shown to reduce infiltration of renal inflammatory cells and downregulate the expression of inflammatory cytokines in kidney tissues in a DKD mouse model, as well as delaying the progression of DKD (11). However, few clinical studies have evaluated the correlation between serum Cat-S and the diagnostic value for DKD.

Vascular injury was among the main mechanisms in the pathogenesis of DKD (12). Therefore, detecting the expression of Cat-S which is a vascular injury marker and determining whether serum Cat-S in T2DM patients is a risk factor for the incidence of DKD are of great significance for preventing the incidence of DKD, strengthening early screening for DKD, and searching for new therapeutic targets for the treatment of DKD in T2DM patients. Therefore, in this study, we detected the expression of serum Cat-S in DKD patients, analyzed the correlation between serum Cat-S and the severity of DKD and analyzed whether serum Cat-S was a risk factor for DKD in T2DM patients, and investigated the diagnostic value for DKD, to provide a theoretical basis for early screening of DKD.

## 2 Materials and methods

### 2.1 Participants

A total of 200 patients with T2DM aged between 18 and 80 years were selected as subjects. A further 43 healthy subjects who underwent physical examination in Henan Provincial People’s Hospital during the same period were selected as the healthy control group, which was matched with the enrolled T2DM participants in gender and age (18 females and 25 males, with an average age of 53.72 ± 9.95 years). The healthy controls had no history of diabetes, kidney disease or any major diseases, and did not meet any of the diagnostic criteria for diabetes. This study was approved by the Ethics Committee of Henan Provincial People’s Hospital [approval number: (2021) No. (153)], and all research subjects signed informed consents.

### 2.2 Inclusion criteria

Patients were eligible for inclusion if they: (1) aged between 18 and 80 years old; (2) met the diagnostic criteria for diabetes mellitus (DM) formulated by the ADA in 2020 (13) and/or the Chinese Guidelines for Clinical Diagnosis and Treatment of Diabetic Kidney Disease in 2021 (14); (3) had complete clinical data available; (4) signed the informed consent form voluntarily.

### 2.3 Exclusion criteria

Participants were excluded if they (1) had other types of diabetes; (2) had diabetes combined with acute or chronic infection; (3) had diabetes combined with acute cardiovascular or cerebrovascular diseases; (4) had diabetes combined with chronic obstructive pulmonary disease and aspartate aminotransferase or alanine transaminase ≥2 times the upper limit of normal simultaneously; (5) had complications involving other renal diseases; (6) were women who were pregnant or lactating; (7) were affected by diabetic ketoacidosis, hypoglycemia coma, lactic acidosis, hyperosmolar coma, surgery, trauma or other stress states; (8) had undergone dialysis or renal transplantation.

### 2.4 Grouping scheme

In grouping mode 1, T2DM patients were divided into three groups according to urine albumin creatinine ratio (UACR) which reflects the severity of albuminuria: T2DM group (UACR <30 mg/g, n=84); early DKD group (UACR 30–300 mg/g, n=41) and clinical DKD group (UACR >300 mg/g, n=75). In grouping mode 2, T2DM patients were divided into two groups according to estimated glomerular filtration rate (eGFR): normal renal function group (eGFR ≥90 mL/min/1.73 m^2^, n=141) and decreased renal function group (eGFR <90mL/min/1.73 m^2^, n=59).

### 2.5 Clinical data collection

The patients’ gender, age, body mass index (BMI), admission systolic blood pressure (SBP), diastolic blood pressure (DBP), fasting blood glucose (FBG), total cholesterol (TC), triglycerides (TG), high-density lipoprotein cholesterol (HDL-C), low-density lipoprotein cholesterol (LDL-C), retinol-binding protein (RBP), blood urea nitrogen (BUN), serum creatinine (Scr), serum uric acid (SUA), CysC and other clinical data were collected. The eGFR was calculated using the Modification of Diet in Kidney Disease equation: eGFR = 186 × age (Scr/88.4) ^-1.154^ × age^-0.203^ (women × 0.742) (15).

### 2.6 Sample collection and Cat-S detection

All patients were required to fast for more than 8 h. In the early morning of the following day, 5 mL of non-anticoagulant venous blood was taken on an empty stomach and allowed to coagulate at room temperature for 2 h. After centrifugation (1000×g for 15 min at 4°C), the serum was collected and stored in a freezer at −80 °C until use. Serum Cat-S concentration (pg/mL) was detected by enzyme-linked immunosorbent assay (ELISA; Wuhan Huamei Biological Engineering, Co., Ltd.) and the operation was carried out strictly according to the instructions. Finally, 5 mL of morning urine was collected for the determination of UACR by immunoturbidimetry.

### 2.7 Statistical analysis

Measurement data conforming to a normal distribution were expressed as mean ± standard deviation ( $\bar{X}\pm \sigma$ ), and measurement data with non-normal distribution were expressed as M (Q1, Q3). As this study involved comparisons of multiple measurement data, a homogeneity of variance test was performed. The homogeneity of variance was analyzed by one-way analysis of variance, and the multiple comparison LSD was used for the comparison between samples. Non-parametric test was used for uneven variance, Kruskal–Wallis test was used for multiple group comparisons, and Bonferroni correction was used for pairwise comparisons. Qualitative data were compared between groups with chi-square test. Spearman rank correlation analysis was used to analyze the correlations between serum Cat-S level and clinical indicators. Multivariate logistic regression was used to analyze the risk factors for DKD in T2DM patients. The AUC (area under the receiver operating characteristic curve (ROC)) was used to analyze the diagnostic value of serum Cat-S and CysC in early DKD. All tests were two-tailed and *P*<0.05 was considered to indicate statistical significance. All statistical analysis were performed by SPSS 25.0.

## 3 Results

### 3.1 Comparison of clinical parameters and serum Cat-S under grouping mode 1

Forty-three healthy subjects (healthy control group), 84 T2DM patients (T2DM group), 41 early DKD patients (early DKD group), and 75 clinical DKD patients (clinical DKD group) were enrolled in this study. There were significant differences in BMI, FBG, HbAlC, SUA, Scr, eGFR, BUN, UACR, TG, TC, HDL-C and LDL-C among the four groups (*P*<0.05). There were no significant difference in the ratio of male to female and age among the four groups (*P*>0.05). There were significant differences in SBP, DBP, CysC and RBP among the T2DM group, early DKD group and clinical DKD group (**Table 1**). Serum Cat-S concentrations in each group were as follows: healthy control group: 312.51 (216.58,476.86) pg/mL; T2DM group: 476.45 (257.98,616.72) pg/mL; early DKD group: 684.61 (468.21,834.20) pg/mL; clinical DKD group: 1688.71 (1236.28, 2227.65) pg/mL. There were significant differences in serum Cat-S concentration among the T2DM group, early DKD group and clinical DKD group (*P*<0.05). The serum Cat-S level in the T2DM group was higher than that in the healthy control group, but the difference was not statistically significant (*P*>0.05) (**Table 1**).

**Table 1:** Comparison of clinical indicators and serum Cat-S under grouping mode 1.

Projects	Healthy control group	T2DM group	Early DKD group	Clinical DKD group	*P Value*
Gender (female/male)^a^	25/18	32/52	14/27	26/49	0.960
Age(year)^b^	53.72 ± 9.95	56.26 ± 10.73	57.78 ± 10.72	53.69 ± 9.71	0.113
BMI(Kg/m^2^)^b^	24.20 (23.00, 25.20)	25.09 (23.44, 27.61)^*^	26.11 (23.84, 28.58)^*^	26.41 (24.22, 29.06)^*^	< 0.001
Duration of DM (years)^c^	0	9.5(4.0~16.75)	9.0(4.0~18.0)	10.0(3.0~16.0)	0.793
SBP (mmHg)^b^	124.6 ± 7.85	131.57 ± 16.67	134.80 ± 14.75	147.15 ± 18.70^$&^	< 0.001
DBP (mmHg)^b^	74.77 ± 8.42	75.31 ± 10.98	77.05 ± 12.43	81.90 ± 3.55^$^	0.001
FBG (mmol/L)^c^	4.91(4.60, 5.15)	7.37(5.89, 9.07)^*^	8.18(6.27, 9.83)^*^	7.79(5.29, 9.17)^*^	< 0.001
HbAlC(mmol/L)^c^	5.40(4.90, 5.60)	8.00(7.23, 9.80)^*^	8.90(7.50,10.65)^*^	8.00(7.18, 9.00) ^*^	< 0.001
SUA (μmol/L)^c^	295(241, 340)	275(227, 357)	280(215, 346)	358 (289, 425) ^*$&^	< 0.001
Scr (μmol/L)^c^	60.0(55.0, 67.0)	57.5(44.0, 64.0)	55.0(48.0, 62.5)	95.0(68.0,156.0)^*$&^	< 0.001
eGFR mL/min/(1.73 m^2^)^c^	111.62 (96.12, 126.74)	132.45 (109.63, 158.73)^*^	135.56 (108.91, 152.09)^*^	66.28 (36.39, 101.24)^*$&^	< 0.001
BUN (mmol/L)^c^	4.86(4.13, 5.82)	5.83(4.87, 7.00)^*^	5.52(4.83, 6.39)	8.97(6.11,12.20)^*$&^	< 0.001
UACR (mg/g)^c^	10.2(6.4, 11.0)	10.0(6.4, 17.1)	92.4(53.9,188.1)^*$^	3271.0(1378.0, 6012.5)^*$&^	< 0.001
TG (mmol/L)^c^	1.20(0.90, 1.58)	1.42(1.08, 2.28)	1.64(1.19, 2.59)^*^	1.81(1.46, 2.36)^*$^	< 0.001
TC (mmol/L)^c^	4.38(3.69, 5.12)	4.58(3.85, 5.46)	4.19(3.49, 5.10)	5.18(4.32, 6.59)^*$&^	< 0.001
HDL-C (mmol/L)^c^	1.37(1.17, 1.52)	1.10(0.98, 1.29)^*^	1.06(0.90, 1.36)^*^	1.08(0.88, 1.31)^*^	< 0.001
LDL-C (mmol/L)^c^	2.92(2.14, 3.59)	2.73(2.10, 3.40)	2.43(1.93, 3.08)	2.88(2.41, 3.96)^&^	0.011
Cat-S (pg/mL)^c^	312.51 (216.58,476.86)	476.45 (257.98,616.72)	684.61 (468.21,834.20)^*$^	1688.71 (1236.28, 2227.65)^*$&^	< 0.001
CysC (mg/L)^c^	–	0.84(0.75, 0.93)	0.88(0.84, 0.96)	1.57(1.04, 2.30)^$&^	< 0.001
RBP (mg/L)^c^	–	38.0(33.9, 46.2)	39.2(33.8, 46.7)	53.9(46.0, 66.3) ^$&^	< 0.001

BMI, body mass index; SBP, systolic blood pressure; DBP, diastolic blood pressure; FBG, fasting blood glucose; HbAlC, glycosylated hemoglobin; SUA, blood uric acid; Scr, serum creatinine; eGFR, estimated glomerular filtration rate; BUN, blood urea nitrogen; UACR, urinary albumin/creatinine ratio; TG, triglyceride; TC, total cholesterol; HDL⁃C, high-density lipoprotein cholesterol; LDL⁃C, low-density lipoprotein cholesterol; Cat-S, Cathepsin S; CysC, cystatin C; RBP, retinol-binding protein; Data are presented as( x ± s)or M (1/4, 3/4) unless indicated; *: P < 0.05 compared with healthy controls; $: Comparison with T2DM group P < 0.05; &: comparison with early DKD group P < 0.05; ^a^ χ2 test; ^b^ one-way analysis of variance; ^c^ rank sum test.

### 3.2 Correlations between serum Cat-S and clinical parameters

The serum Cat-S level was positively correlated with UACR (r=0.76), RBP, BUN, Scr, TC, SUA, CysC (r=0.548) (*P*<0.05) and was negatively correlated with eGFR (r=−0.54) (*P*<0.001). The serum CysC level was positively correlated with UACR (r=0.604), RBP, BUN, Scr, TG, SUA, Cat-S (r=0.548) (*P*<0.05) and was negatively correlated with eGFR (r=−0.779) (*P*<0.001) (**Table 2**).

**Table 2:** Spearman analysis between serum Cat-S, CysC and clinical parameters (n=200).

Projects	Cat-S		CysC	
	r	*P Value*	r	*P Value*
UACR (mg/g)	0.76	< 0.001	0.604	< 0.001
eGFR mL/min/(1.73 m^2^)	-0.54	< 0.001	-0.779	< 0.001
RBP (mg/L)	0.38	< 0.001	0.610	< 0.001
BUN (mmol/L)	0.40	< 0.001	0.609	< 0.001
Scr (μmol/L)	0.53	< 0.001	0.749	< 0.001
FBG (mmol/L)	-0.08	0.28	-0.127	0.074
TC (mmol/L)	0.14	0.04	0.108	0.126
TG (mmol/L)	0.12	0.09	0.216	0.002
LDL-C (mmol/L)	0.07	0.35	0.076	0.282
HDL-C (mmol/L)	-0.08	0.27	-0.159	0.025
SUA (μmol/L)	0.25	< 0.001	0.451	< 0.001
Cat-S (pg/mL)	–	–	0.548	< 0.001
CysC (mg/L)	0.548	< 0.001	–	–

### 3.3 Univariate logistic regression analysis of all parameters under grouping mode 1

Taking the occurrence of DKD or not as the dependent variable (assignment: T2DM=1, early DKD=2, clinical DKD=3) and SBP, DBP, SUA, TC, TG, LDL-C, eGFR, BUN, RBP, Cat-S and CysC as the independent variables. Univariate logistic regression analysis showed that: Cat-S may be an influential factor on the occurrence of early DKD (*P*<0.001); and SBP, DBP, SUA, TC, LDL-C, eGFR, BUN, RBP, Cat-S and CysC may be influential factors on the occurrence of clinical DKD (*P*<0.01) (**Table 3**).

**Table 3:** Univariate Logistic regression analysis of all parameters under grouping mode 1.

Independent variable	Eraly DKD		Clinical DKD	
	*OR (95%CI)*	*P Value*	*OR (95%CI)*	*P Value*
SBP (mmHg)	1.012(0.989~1.036)	0.298	1.055(1.033~1.078)	< 0.001
DPB (mmHg)	1.01(0.981~1.049)	0.409	1.05(1.023~1.086)	0.001
SUA (μmol/L)	0.99(0.993~1.003)	0.377	1.00(1.004~1.012)	< 0.001
TC (mmol/L)	0.86(0.633~1.190)	0.378	1.59(1.243~2.051)	< 0.001
TG (mmol/L)	1.02(0.846~1.238)	0.810	1.01(0.857~1.193)	0.898
LDL-C (mmol/L)	0.76(0.506~1.160)	0.202	1.58(1.157~2.161)	0.004
eGFR mL/min/(1.73 m^2^)	0.99(0.989~1.007)	0.661	0.96(0.949~0.971)	< 0.001
BUN (mmol/L)	0.88(0.712~1.102)	0.278	1.50(1.285~1.762)	< 0.001
RBP (mg/L)	0.98(0.952~1.010)	0.194	1.04(1.020~1.067)	< 0.001
Cat-S (0.1ng/dL)	1.50(1.242~1.814)	< 0.001	2.90(2.131~3.957)	< 0.001
CysC (10mg/L)	1.28(0.997~1.648)	0.053	2.09(1.619~2.722)	< 0.001

### 3.4 Multivariate logistic regression analysis of serum Cat-S and CysC under grouping mode 1

The data of this study did not meet the requirements of the ordered logistic regression parallelism test, so multivariate logistic regression analysis was used to analyze the risk factors for early DKD and clinical DKD. Taking whether DKD occurs or not as the dependent variable (assignment: T2DM=1, early DKD=2, clinical DKD=3), the results showed that after correcting SBP, DBP, SUA, TC, eGFR, BUN, RBP and other factors, we found that Cat-S and CysC were independent risk factors for DKD and clinical DKD in T2DM patients (*P*<0.05). For every 0.1 unit increase in Cat-S, the relative risks of early DKD and clinical DKD in T2DM patients increased by 1.541 times and 5.690 times, respectively. For every 10 units increase in CysC, the relative risks of early DKD and clinical DKD in T2DM increased by 1.611 and 2.880 times, respectively (**Table 4**).

**Table 4:** Multivariate Logistic regression of Cat-S and CysC under grouping mode 1.

	serum Cat-S (0.1ng/L)		serum CysC (10mg/L)	
	*OR (95%CI)*	*P Value*	*OR (95%CI)*	*P Value*
T2DM	1		1	
Eraly DKD	1.541(1.244~1.910)	<0.001	1.611(1.041~2.492)	0.032
Clinical DKD	5.69(1.856~17.506)	0.002	2.880(1.175~7.062)	0.021

### 3.5 Logistic regression analysis of serum Cat-S on DKD

When UACR ≥ 30 mg/g was defined as the standard for the diagnosis of DKD, UACR < 30 mg/g was defined as DKD (-) group, while UACR ≥ 30 mg/g was defined as DKD (+) group. Taking whether DKD occurs or not as the dependent variable (assignment: DKD (-) =1, DKD (+) =2) and Age, Weight, Hight, SBP, DSP, Cat-S, BMI, HbAlC, eGFR, BUN, SUA, FBG, CysC, RBP, TC, TG, LDL-C, LDL-C and other factors as independent variables, the univariate logistic regression analysis showed that SBP, DSP, eGFR, BUN, SUA, RBP, TC, Cat-S, CysC may be the influencing factors of DKD (+) (P < 0.05) (**Table 2**). Taking whether DKD occurs or not as the dependent variable (assignment: DKD (-) =1, DKD (+) =2), the multivariate logistic regression analysis was performed and the results showed: after adjusting for SBP, DSP, eGFR, BUN, SUA, RBP, TC and other factors, we found the increased Cat-S and CysC were risk factors for the onset of DKD (P < 0.05). The relative risk of DKD in T2DM patients increased by 1.626 times for every 0.1 unit increase in Cat-S. For every 10 units of CysC, the relative risk of DKD in T2DM increased by 1.657 times (**Table 5**).

**Table 5:** Multivariate logistic regression analysis of serum Cat-S on DKD.

Independent variable	Univariate		Multivariate	
	*OR*	*P Value*	*OR*	*P Value*
SBP (mmHg)	1.038(1.019, 1.057)	<0.001	1.004(0.972, 1.037)	0.824
DSP (mmHg)	1.039(1.012, 1.067)	0.04	1.030(0.986, 1.076)	0.180
eGFR mL/min/(1.73 m^2^)	0.980(0.973, 0.987)	<0.001	1.004(0.990, 1.018)	0.588
BUN (mmol/L)	1.278(1.129, 1.447)	<0.001	0.825(0.627, 1.084)	0.167
SUA (μmol/L)	1.005(1.001, 1.008)	0.006	0.999(0.994, 1.005)	0.821
RBP (mg/L)	1.022(1.002, 1.042)	0.027	0.995(0.975, 1.016)	0.657
TC (mmol/L)	1.304(1.056, 1.610)	0.014	1.180(0.820, 1.699)	0.374
Cat-S (0.1 ng/dL)	1.700(1.435, 2.014)	<0.001	1.626(1.335, 1.981)	<0.001
CysC (10 mg/L)	1.640(1.339, 2.009)	<0.001	1.657(1.111, 2.470)	0.013

### 3.6 Diagnostic value of serum Cat-S and CysC in clinical DKD under grouping mode 1

**Figure 1:**
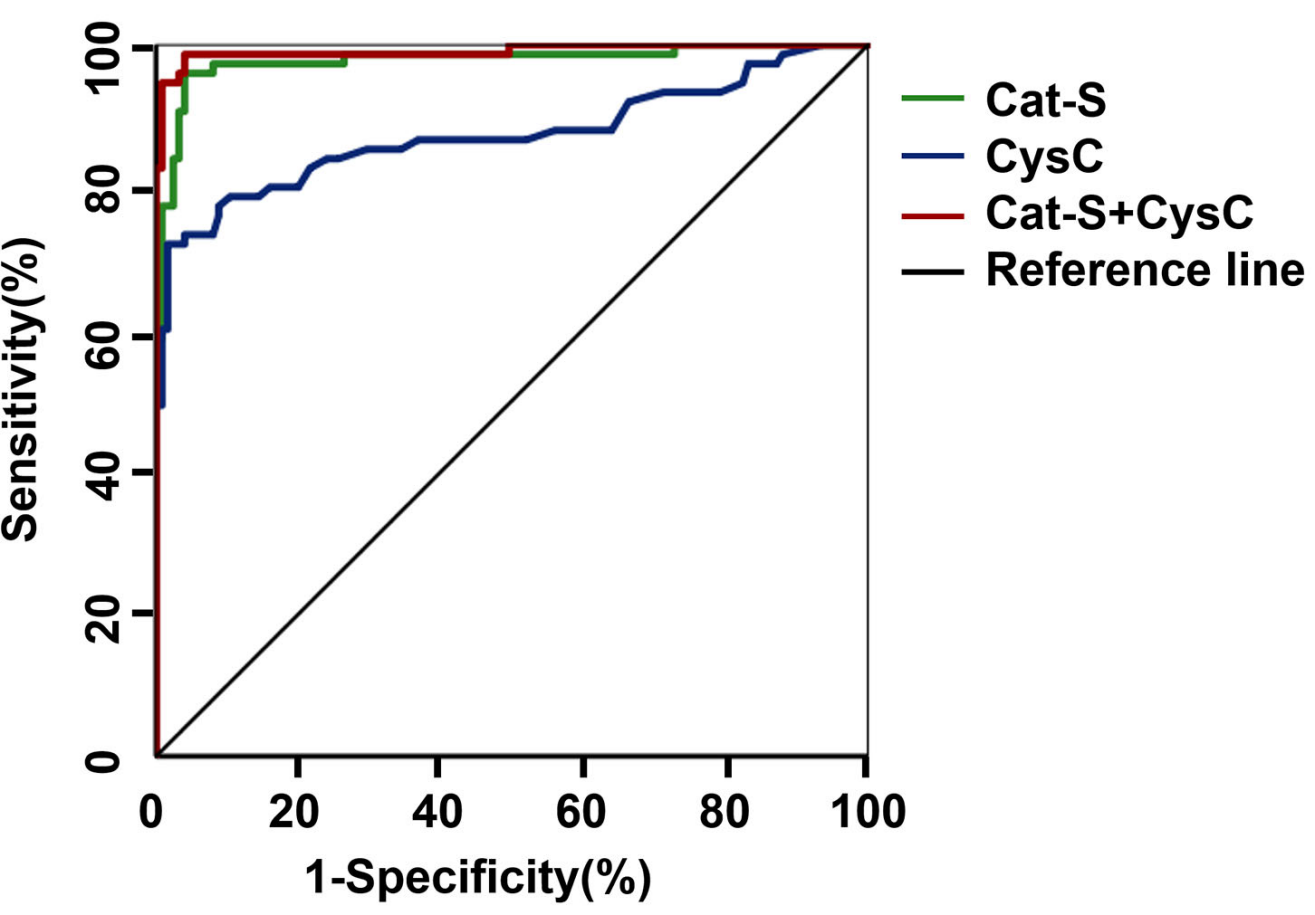
ROC curve of serum Cat-S and CysC in diagnosing clinical DKD. Cat-S, Cathepsin S; CysC, cystatin C.

UACR >300 mg/g was defined as the standard for the diagnosis of clinical DKD. The ROC curve analysis gave a result of AUC which was 0.978 for serum Cat-S for diagnosing clinical DKD, and when the cut-off value of serum Cat-S was 974.14 pg/mL, the sensitivity was 96% and the specificity was 96% for diagnosing clinical DKD (*P*<0.001). While the AUC of CysC was 0.874 for diagnosing clinical DKD, and when the cut-off value of CysC was 1.16 mg/L, the sensitivity was 72% and the specificity was 98% (*P*<0.001). Results indicated that serum Cat-S was superior to CysC in the diagnosis of clinical DKD. The AUC for the combined diagnosis of clinical DKD was 0.991, the sensitivity increased to 98% and the specificity was 96% (**Figure 1** and **Table 6**).

**Table 6:** The diagnostic value of serum Cat-S and CysC in clinical DKD under grouping mode 1.

Subjects	AUC	*P Value*	*95%CI*	optimal cut-off value	Sensitivity%	Specificity%	youden index
Cat-S	0.978	< 0.001	0.957~1	974.14pg/mL	0.96	0.96	0.920
CysC	0.874	< 0.001	0.814~0.934	1.16mg/L	0.72	0.98	0.704
Cat-S+CysC	0.991	< 0.001	0.978~1	–	0.98	0.96	0.947

### 3.7 Diagnostic value of serum Cat-S and CysC in DKD under grouping mode 1

**Figure 2:**
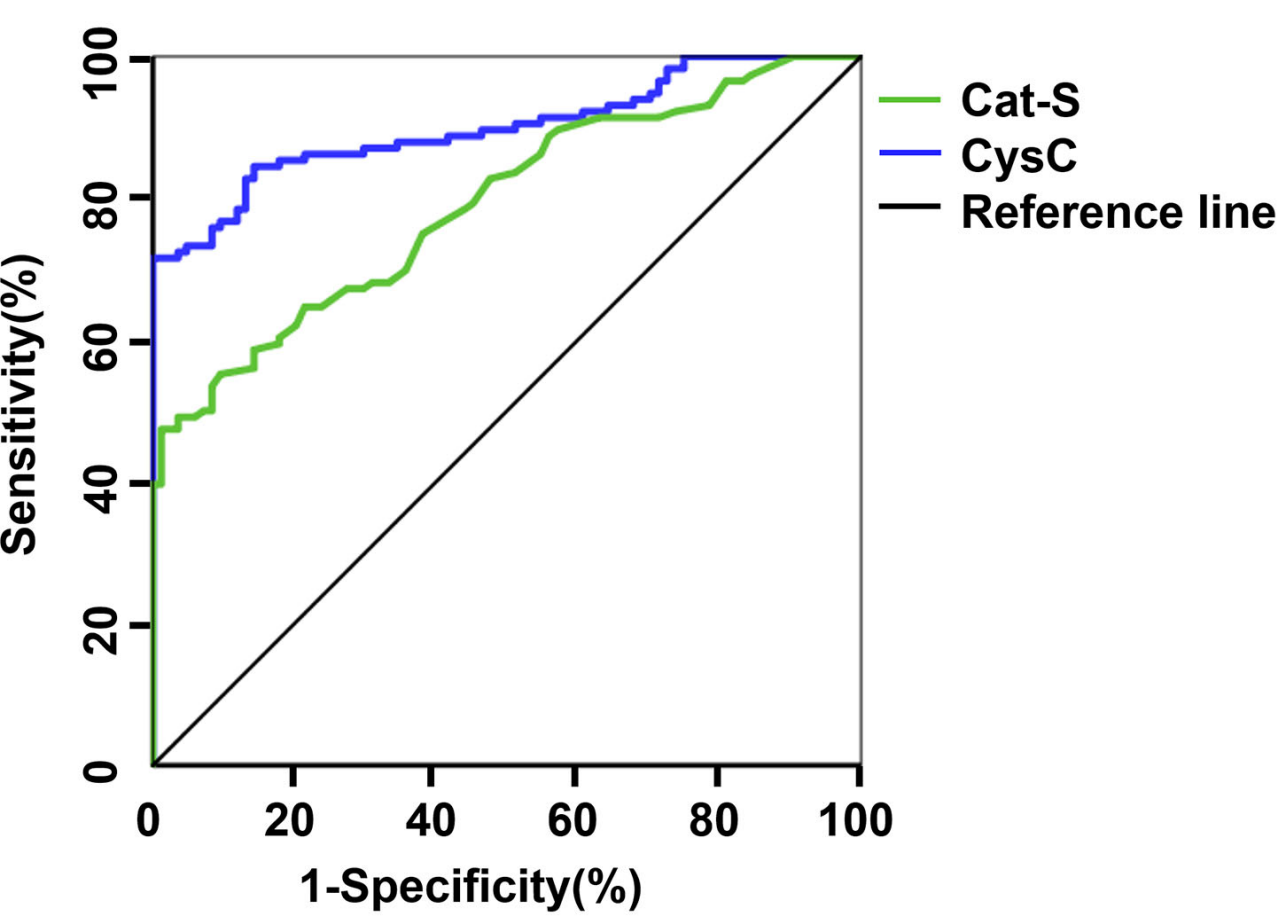
ROC curve of serum Cat-S and CysC in diagnosing DKD. Cat-S, Cathepsin S; CysC, cystatin C.

Defining UACR ≥30 mg/g as the standard for the diagnosis of early DKD, we assessed the diagnostic value of serum Cat-S and CysC based on the AUC of the ROC curve. The AUC for Cat-S in the diagnosis of DKD was 0.900, the cut-off value was 827.42 pg/mL, and the sensitivity and specificity were 71.6% and 98.8%, respectively. The AUC for CysC in the diagnosis of DKD was 0.791, and, when the cut-off value was 1.16mg/L, the sensitivity and specificity of CysC in the diagnosis of DKD were 47.4% and 98.8%, respectively (**Figure 2** and **Table 7**).

**Table 7:** The diagnostic value of serum Cat-S and CysC in DKD under grouping mode 1.

Subjects	AUC	*P Value*	*95%CI*	optimal cut-off value	Sensitivity%	Specificity%	youden index
Cat-S	0.900	< 0.001	0.858~0.943	827.42pg/mL	0.716	0.988	0.706
CysC	0.791	< 0.001	0.731~0.852	1.16mg/L	0.474	0.988	0.462

### 3.8 Comparison of clinical parameters and serum Cat-S under grouping mode 2

There was no significant difference in gender, age, BMI, FBG or HDL-C between the normal renal function group and decreased renal function group (*P*>0.05). The SBP, DBP, SUA, Scr, BUN, UACR, TC, TG, LDL-C, Cat-S and CysC in the normal renal function group were significantly lower than those in the decreased renal function group (*P*<0.05) (**Table 8**).

**Table 8:** Comparison of clinical indicators and serum Cat-S under grouping mode 2.

Projects	Normal renal function group (n=141)	Decreased renal function group (n=59)	*Z/χ^2^/t*	*P Value*
Gender(female/male) ^a^	47/94	25/34	1.475	0.225
Age (year) ^b^	55.29 ± 10.56	56.32 ± 10.21	0.636	0.526
BMI (Kg/m^2^) ^b^	25.94 ± 3.33	26.73 ± 3.92	1.447	0.149
SBP (mmHg) ^b^	133.37 ± 15.75	149.32 ± 19.68	5.528	< 0.001
DBP (mmHg) ^b^	76.89 ± 10.71	81.12 ± 13.03	2.383	0.018
FBG (mmol/L) ^c^	7.45 (5.98, 9.33)	7.00 (5.28, 9.29)	-1.421	0.155
HbAlC (mmol/L) ^c^	8.40 (7.25, 9.90)	8.00 (7.35, 8.90)	-1.644	0.100
SUA (μmol/L) ^c^	281.0 (228.5, 347.0)	374.0 (323.0, 447.0)	-5.754	< 0.001
Scr (μmol/L) ^c^	57.0(46.0, 64.0)	124.0 (85.0, 193.0)	-10.663	< 0.001
eGFR mL/min/(1.73 m^2^) ^c^	131.75 (110.42, 158.49)	48.66 (28.88, 71.84)	-11.143	< 0.001
BUN (mmol/L) ^c^	5.65 (4.87, 6.71)	9.57 (7.65, 13.58)	-8.388	< 0.001
UACR (mg/g) ^c^	26.30(9.85, 172.00)	3896.70(1487.40, 6345.60)	-8.503	< 0.001
TC (mmol/L) ^c^	4.57 (3.72, 5.44)	5.20 (4.32, 6.62)	-3.682	< 0.001
TG (mmol/L) ^c^	1.52 (1.18, 2.27)	2.00 (1.46, 2.59)	-2.644	0.008
HDL-C (mmol/L) ^c^	1.08 (0.96, 1.31)	1.09 (0.91, 1.31)	-0.441	0.659
LDL-C (mmol/L) ^c^	2.67 (2.02, 3.29)	3.13 (2.49, 4.04)	-3.338	0.001
Cat-S (pg/mL) ^c^	594.24(374.05, 787.46)	1642.78(1107.57, 2192.67)	-7.889	< 0.001
CysC (mg/L) ^c^	0.87 (0.78, 0.96)	1.78 (1.25, 2.37)	-9.740	< 0.001
RBP (mg/L) ^c^	39.0 (33.8, 47.0)	58.8 (47.3, 71.3)	-7.823	< 0.001

normal renal function group (eGFR≥90mL/min/1.73 m^2^); decreased renal function group (eGFR<; 90mL/min/1.73 m^2^); ^a^ χ2 test; ^b^ one-way analysis of variance; ^c^ rank sum test.

### 3.9 Logistics regression analysis of renal function decline in T2DM patients under grouping mode 2

Taking whether eGFR declined or not as the dependent variable (assignment: eGFR ≥90 mL/min/(1.73 m^2^)=0, eGFR <90 mL/min/(1.73 m^2^)=1) and SBP, DBP, SUA, BUN, UACR, TC, LDL-C, Cat-S, CysC and RBP as independent variables, univariate logistic regression analysis was performed using the “enter” method. The results identified SBP, DBP, SUA, BUN, UACR, TC, LDL-C, Cat-S, CysC and RBP as potential risk factors for eGFR <90mL/min/(1.73 m^2^) (*P*<0.05). We took the significantly meaningful variables (SBP, DBP, SUA, BUN, UACR, TC, LDL-C, Cat-S, CysC and RBP) from the univariate logistic regression analysis as independent variables for multivariate logistic regression analysis by the “backward: LR” method. The results showed that increased SBP (odds ratio (OR)=1.044, 95% CI=1.004–1.087, *P*=0.033), increased BUN (OR=1.674, 95% CI=1.228–2.280, *P=*0.001), increased TC (OR=1.956, 95% CI=1.324–2.889, *P*=0.001), increased Cat-S (OR=2.835, 95% CI=1.260–6.381, *P*=0.012) and elevated CysC (OR=1.345, 95% CI=1.116–1.620, *P*=0.002) were independent risk factors for eGFR <90 mL/min/(1.73 m^2^). Moreover, the relative risk of eGFR <90 mL/min/(1.73 m^2^) increased with increasing Cat-S concentration; that is, the higher the levels of SBP, BUN, TC, Cat-S and CysC, the higher the probability of eGFR <90 mL/min/(1.73 m^2^) (**Table 9**).

**Table 9:** Logistics regression analysis of renal function decline in T2DM under grouping mode 2.

Independent variable	Univariate		Multivariate	
	*OR*	*P Value*	*OR*	*P Value*
SBP (mmHg)	1.055 (1.034~1.077)	< 0.001	1.044 (1.004~1.087)	0.033
DBP (mmHg)	1.033 (1.005~1.061)	0.02	0.943 (0.885~1.003)	0.064
SUA (μmol/L)	1.011 (1.007~1.015)	< 0.001	1.006 (0.999~1.014)	0.093
BUN (mmol/L)	1.949 (1.589~2.390)	< 0.001	1.674 (1.228~2.280)	0.001
UACR (mg/g)	1.001 (1.001~1.001)	< 0.001	–	–
TC (mmol/L)	1.630 (1.288, 2.063)	< 0.001	1.956 (1.324~2.889)	0.001
LDL-C (mmol/L)	1.855 (1.359~2.531)	< 0.001	–	–
Cat-S (0.1ng/mL)	5.501 (3.264~9.272)	< 0.001	2.835 (1.260~6.381)	0.012
CysC (10mg/L)	1.799 (1.489~2.174)	< 0.001	1.345 (1.116~1.620)	0.002
RBP (mg/L)	1.058 (1.034~1.082)	< 0.001	–	–

### 3.10 Diagnostic value of serum Cat-S and CysC in T2DM with decreased renal function

**Figure 3:**
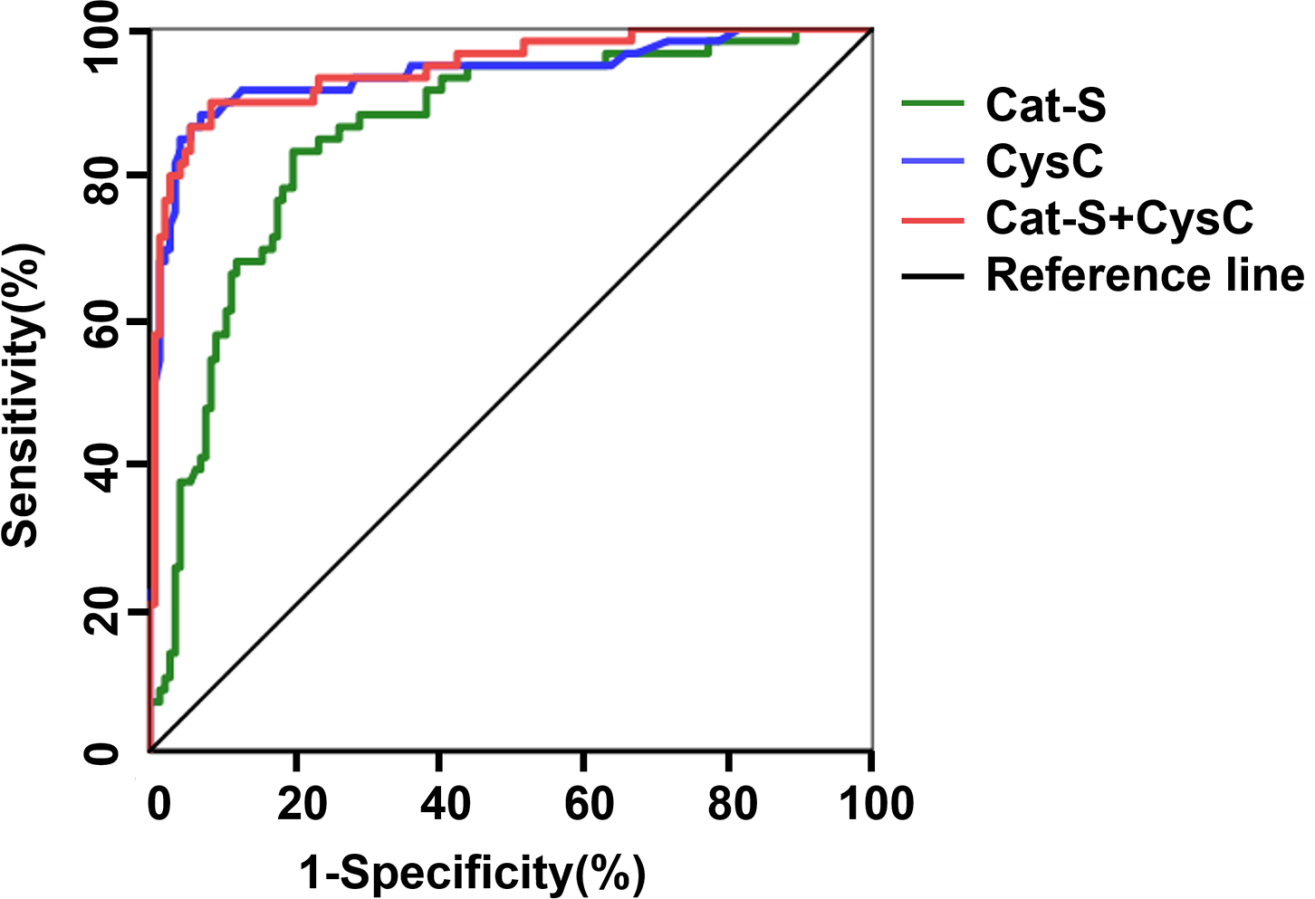
ROC curve of serum Cat-S and CysC in diagnosing renal function decline in T2DM.

When eGFR <90 mL/min/(1.73 m^2^) was defined as the diagnostic criterion for renal function decline. The ROC analysis gave an AUC of 0.854 for serum Cat-S in diagnosing decreased renal function of DKD, and when the cut-off value of serum Cat-S was 974.14 pg/mL, the sensitivity and specificity of serum Cat-S in diagnosing DKD were 85% and 80% respectively. While the AUC of serum CysC was 0.937 for diagnosing decreased renal function of DKD; and when the cut-off value of serum CysC was 1.08mg/L, the sensitivity and specificity of serum CysC in the diagnosis of decreased renal function were 88% and 93%, respectively. These results indicated that CysC was superior to serum Cat-S in diagnosing renal function decline (eGFR <90mL/min/(1.73 m^2^)) in T2DM patients. The combination of serum CysC and Cat-S in the diagnosis of renal function decline showed an improved diagnostic value, with the AUC increased to 0.945, the sensitivity to 90% and the specificity to 85% compared with diagnosing by serum Cat-S only (**Table 10** and **Figure 3**).

**Table 10:** The diagnostic value of serum Cat-S and CysC in T2DM with renal function decline.

Subjects	AUC	*P Value*	95%CI	optimal cut-off value	Sensitivity	Specificity	youden index
Cat-S	0.854	< 0.001	0.797~0.911	974.14pg/mL	0.85	0.80	0.632
CysC	0.937	< 0.001	0.893~0.981	1.08mg/L	0.88	0.93	0.810
Cat-S+CysC	0.945	< 0.001	0.909~0.982	–	0.90	0.85	0.813

## 4 Discussion

DKD is characterized by persistent increased albuminuria excretion and/or progressive decline in renal function, which eventually progress to ESRD and severely affects the quality of life of patients (16). We found that elevated serum Cat-S was associated with the progression of albuminuria and decreased renal function in T2DM patients and could be used to assess the severity of DKD, and presented better diagnostic value than traditional biomarker CysC for diagnosing DKD. These results lay a foundation for the exploration of novel biomarkers for diagnosing early DKD and also indicate potential possibility for the prevention of DKD, early diagnosis, early treatment, as well as improving prognosis of patients.

The pathogenesis of DKD was complex and involved multiple pathways including injury of glomerular endothelial cells, activation of transforming growth factor-β1 and inflammatory responses (17). Cat-S was a proteolytic enzyme that remains active in both acidic and neutral environments, and injection of recombinant Cat-S has been shown to damage glomerular endothelial cells to induce proteinuria and glomerulosclerosis in DKD mice (11). Cat-S was also found to promote renal fibrosis by regulating the TGF-β1/Smad pathway in TGF-β1-stimulated renal tubular epithelial cells, which resulting in decreased renal function (18). In addition, Cat-S promoted the secretion of pro-inflammatory cytokines such as tumor necrosis factor-α and interleukin-1 and activated ELR + CXC chemokines, thereby recruiting inflammatory cells such as macrophages, which secreted Cat-S to exacerbate the inflammatory response in turn (19, 20). The above results suggest that Cat-S is involved not only in dysfunction of glomerular endothelial cells and injury of glomerular filtration barrier that leading to proteinuria, but also in renal fibrosis and inflammatory response which promoting the development of DKD and leading to the decline in renal function. The relationship between Cat-S and DKD has been limited to fundamental research, and few clinical studies have evaluated the relationship between serum Cat-S and DKD in patients with T2DM. We grouped T2DM patients according to UACR and eGFR to investigate the correlation between serum Cat-S level and the severity of DKD, and to assess the diagnostic value of serum Cat-S for diagnosing DKD and the role of serum Cat-S in the assessment of renal function.

Serum Cat-S levels in T2DM patients were closely related to the severity of DKD. In this study, DKD was staged according to UACR which reflected the level of albuminuria in T2DM patients. The results showed that the level of serum Cat-S in patients with different DKD stages tended to increase with the increasing UACR; that is, the serum Cat-S level was positively correlated with the level of UACR. A study involving 103 DM patients found that serum Cat-S levels were higher in DM patients than in healthy control group (21), which is consistent with the results of our study, but they did not analyze the relationship between serum Cat-S and the severity of DKD. T2DM patients were further grouped according to eGFR. We found serum Cat-S was negatively correlated with eGFR, indicating that the increase of serum Cat-S was concurrent with the decrease of renal function. Serum Cat-S has been found to be negatively correlated with eGFR in German chronic kidney disease (CKD) patients and Swedish community CKD patients (22), which were consistent with the results of this study. These results suggest that serum Cat-S in T2DM patients increases with the progression of DKD. Thus, serum Cat-S is expected to applicate as a novel biomarker for the early diagnosis of clinical DKD.

We grouped T2DM patients according to UACR which reflected the severity of albuminuria and according to eGFR, and found that the elevated serum Cat-S and CysC were independent risk factors for early DKD and decreased renal function in T2DM patients. Multivariate logistic regression analysis under grouping mode 1 showed that with an increase of 0.1 units of Cat-S, the relative risks of early DKD and clinical DKD increased 1.541 times and 5.690 times, respectively; whereas with an increase of 10 units of CysC, the relative risks of early DKD and clinical DKD increased by 1.611 times and 2.880 times, respectively. Multivariate logistic regression analysis under grouping mode 2 showed that elevated serum Cat-S and CysC were independent risk factors for eGFR < 90mL/min/(1.73 m^2^); that is, the higher the serum Cat-S and CysC levels, the higher the probability of eGFR < 90mL/min/(1.73 m^2^). In db/db mice with type 2 diabetes, Cat-S inhibitors or PAR2 inhibitors have been reported to reduce albuminuria and glomerulosclerosis, as well as other organ complications such as diabetic retinopathy (11). Therefore, we conclude that serum Cat-S was associated with the development of increased albuminuria and decreased renal function, and could be a potential new therapeutic target for the prevention of DKD.

Serum Cat-S performed valuable diagnostic efficacy in the diagnosis of DKD. Previous studies have suggested that serum CysC in T2DM patients was positively correlated with UACR (23), which was a sensitive indicator of renal impairment and could be used as a marker for early diagnosis of DKD (24). However, in this study, serum CysC increased significantly in T2DM patients with massive albuminuria, which indicating that serum CysC could not be used to diagnose early DKD effectively. Therefore, we further analyzed and compared the diagnostic value of serum Cat-S and serum CysC in DKD. When UACR ≥30mg/g was used as the criterion for the diagnosis of early DKD, ROC analyzed the diagnostic value of serum Cat-S showed that when the optimal cut-off value for serum Cat-S was 827.42 pg/mL, the diagnostic sensitivity and specificity of serum Cat-S were higher than those of serum CysC, suggesting that serum Cat-S has better diagnostic efficacy for the diagnosis of DKD. When eGFR < 90mL/min/(1.73 m^2^) was used as the diagnostic criterion for decreased renal function, we found that serum Cat-S and serum CysC had similar diagnostic value for decreased renal function in T2DM patients. However, the combined diagnostic value of serum Cat-S and serum CysC for diagnosing decreased renal function in patients with T2DM was better than that of either of the two alone, suggesting that serum Cat-S level could reflect the decline of renal function of T2DM patients to a certain extent.

However, this study had some limitations. However, this study had some limitations. Researchers have found that strict control of blood glucose can delay the progression of DKD in T2DM patients (25). Considering that diabetes medications may be used, there were no differences in HbAlC between subgroups in this study. We did not count the application of hypoglycemic drugs, but it will be a new direction in our following study. Patney et al. demonstrated that hypertension accelerated the progression of renal disease and led to increased morbidity and mortality from cardiovascular complications in DKD patients (26). We found that elevated SBP and DBP were influential factors in the occurrence of early DKD and decreased renal function. Therefore, controlling cardiovascular complications such as blood pressure may be one of the directions to slow the progression of DKD. It will be an important direction to research DKD with other cardiovascular complications. In the follow-up study, we will explore the diagnostic value of Cat-S for diagnosing DKD with or without cardiovascular complications. It was a single-center cross-sectional study, and further verification is needed in a multi-center prospective study with a large sample size and we plan to follow up DKD patients to analyze the correlation between Cat-S and the prognosis of DKD. In addition, most DKD patients we enrolled didn’t undergo renal biopsy, thus the relationship between Cat-S and renal pathology was not clarified in our study. It was a single-center cross-sectional study, and further verification is needed in a multi-center prospective study with a large sample size. In addition, most DKD patients were clinically confirmed but not pathologically confirmed because clinically confirmed DKD patients usually did not undergo renal biopsy, thus the relationship between Cat-S and renal pathology was not clarified in our study.

## 5 Conclusion

In conclusion, we concluded that the elevated serum Cat-S were associated with the progression of albuminuria and decreased renal function in T2DM patients. Serum Cat-S increased with the severity of albuminuria in patients with DKD, and the diagnostic value of serum Cat-S was better than that of CysC for diagnosing DKD. The level of serum Cat-S could reflect the decline of renal function in T2DM patients to a certain extent. As a risk factor that affects the incidence of DKD, serum Cat-S is expected to be a new biomarker for the early diagnosis and severity assessment of DKD.

## Data availability statement

The raw data supporting the conclusions of this article will be made available by the authors, without undue reservation.

## Ethics statement

The studies involving human participants were reviewed and approved by the Ethics Committee of Henan Provincial People’s Hospital [Approval number (2021): No (153)]. The patients/participants provided their written informed consent to participate in this study.

## Author contributions

FS designed the study, WW collected the data, XR and WW analyzed the data. XR wrote the manuscript. All authors contributed to the article and approved the submitted version.

## References

[1] Conserva F Gesualdo L Papale M. A systems biology overview on human diabetic nephropathy: from genetic susceptibility to post-transcr iptional and post-translational modifications. J Diabetes Res (2016) 2016:7934504. doi: 10.1155/2016/7934504 26798653 PMC4698547

[2] Zhang D Ye S Pan T. The role of serum and urinary biomarkers in the diagnosis of early diabetic nephropathy in patients with type 2 diabetes. PeerJ (2019) 7:e7079. doi: 10.7717/peerj.7079 31218128 PMC6568248

[3] Li WN Wang JL Ge LN Shan JH Zhang CB Liu JP. Growth arrest-specific protein 6 (Gas6) as a noninvasive biomarker for early detection of diabetic nephropathy. Clin Exp Hypertension (2017) 39(4):382–7. doi: 10.1080/10641963.2017.1288739 28513288

[4] Arceo ES Dizon GA Tiongco REG. Serum cystatin c as an early marker of nephropathy among type 2 diabetics: a meta-analysis. Diabetes Metab Syndr (2019) 13(6):3093–7. doi: 10.1016/j.dsx.2019.11.007 31778883

[5] Nikolic-Paterson DJ. Cathepsin s-dependent protease-activated receptor-2 activation: a new mechanism of endothelial dysfunction. J Am Soc Nephrol (2016) 27(6):1577–9. doi: 10.1681/ASN.2015101162 PMC4884124 26590253

[6] Figueiredo JL Aikawa M Zheng C Aaron J Lax L Libby P. Selective cathepsin s inhibition attenuates atherosclerosis in apolipoprotein e-deficient mice with chronic renal disease. Am J Pathol (2015) 185(4):1156–66. doi: 10.1016/j.ajpath.2014.11.026 PMC4380840 25680278

[7] Wilkinson RD Williams R Scott CJ Burden RE. Cathepsin s: therapeutic, diagnostic, and prognostic potential. Biol Chem (2015) 396(8):867–82. doi: 10.1515/hsz-2015-0114 25872877

[8] Zhao JY Yang YC Wu YB. The clinical significance and potential role of cathepsin s in IgA nephropathy. Front Pediatrics (2021) 9:10. doi: 10.3389/fped.2021.631473 PMC8071879 33912521

[9] Rupanagudi KV Kulkarni OP Lichtnekert J Darisipudi MN Mulay SR Schott B. Cathepsin s inhibition suppresses systemic lupus erythematosus and lupus nephritis because cathepsin s is essential for MHC class II-mediated CD4 T cell and b cell priming. Ann Rheumatic Diseases (2015) 74(2):452–63. doi: 10.1136/annrheumdis-2013-203717 24300027

[10] Jobs E Risérus U Ingelsson E Sundström J Jobs M Nerpin E. Serum cathepsin s is associated with decreased insulin sensitivity and the development of type 2 diabetes in a community-based cohort of elderly men. Diabetes Care (2013) 36(1):163–5. doi: 10.2337/dc12-0494 PMC3526243 22923671

[11] Kumar Vr S Darisipudi MN Steiger S Devarapu SK Tato M Kukarni OP. Cathepsin s cleavage of protease-activated receptor-2 on endothelial cells promotes microvascular diabetes complications. J Am Soc Nephrol (2016) 27(6):1635–49. doi: 10.1681/ASN.2015020208 PMC4884104 26567242

[12] Thomas MC Brownlee M Susztak K Sharma K Jandeleit-Dahm KA Zoungas S. Diabetic kidney disease. Nat Rev Dis Primers (2015) 1:15018. doi: 10.1038/nrdp.2015.18 27188921 PMC7724636

[13] American Diabetes A. 2. classification and diagnosis of diabetes: standards of medical care in diabetes-2020. Diabetes Care (2020) 43(Suppl 1):S14–31. doi: 10.2337/dc20-S002 31862745

[14] Expert group of Chinese Society of Nephrology. Chinese Guidelines for clinical diagnosis and treatment of diabetic kidney disease. Chin J Nephrol (2021) 37(3):255–304. doi: 10.3760/cma.j.cn441217-20201125-00041

[15] Ma YC Zuo L Chen JH Luo Q Yu XQ Li Y. Modified glomerular filtration rate estimating equation for Chinese patients with chronic kidney disease. J Am Soc Nephrol JASN (2006) 17(10):2937–44. doi: 10.1681/ASN.2006040368 16988059

[16] Papadopoulou-Marketou N Kanaka-Gantenbein C Marketos N P. Chrousos G Papassotiriou. I. Biomarkers of diabetic nephropathy: a 2017 update. Crit Rev Clin Lab Sci (2017) 54(5):326–42. doi: 10.1080/10408363.2017.1377682 28956668

[17] Gembillo G Ingrasciotta Y Crisafulli S Luxi N Siligato R Santoro D. Kidney disease in diabetic patients: from pathophysiology to pharmacological aspects with a focus on therapeutic inertia. Int J Mol Sci (2021) 22(9):4824. doi: 10.3390/ijms22094824 34062938 PMC8124790

[18] Zhang X Zhou Y Yu X Huang Q Fang W Li J. Differential roles of cysteinyl cathepsins in TGF-beta signaling and tissue fibrosis. iScience (2019) 19:607–22. doi: 10.1016/j.isci.2019.08.014 PMC6715892 31446224

[19] Repnik U Starr AE Overall CM Turk B. Cysteine cathepsins activate ELR chemokines and inactivate non-ELR chemokines. J Biol Chem (2015) 290(22):13800–11. doi: 10.1074/jbc.M115.638395 PMC4447957 25833952

[20] Klinngam W Fu R Janga SR Edman MC Hamm-Alvarez SF. Cathepsin s alters the expression of pro-inflammatory cytokines and MMP-9, partially through protease -activated receptor-2, in human corneal epithelial cells. Int J Mol Sci (2018) 19(11):3530. doi: 10.3390/ijms19113530 30423938 PMC6274678

[21] Liu J Ma L Yang J Ren A Sun Z Yan G. Increased serum cathepsin s in patients with atherosclerosis and diabetes. Atherosclerosis (2006) 186(2):411–9. doi: 10.1016/j.atherosclerosis.2005.08.001 16140306

[22] Steubl D Kumar SV Tato M Mulay SR Larsson A Lind L. Circulating cathepsin-s levels correlate with GFR decline and sTNFR1 and sTNFR2 levels in mice and humans. Sci Rep (2017) 7:43538. doi: 10.1038/srep43538 28240259 PMC5327444

[23] Siddiqi Z Karoli R Kaul A Fatima J Varshney S Beg MS. Evaluation of neutrophil gelatinase-associated lipocalin and cystatin c as early markers of diabetic nephropathy. Ann Afr Med (2017) 16(3):101–6. doi: 10.4103/aam.aam_12_17 PMC5579892 28671149

[24] Stankute I Radzeviciene L Monstaviciene A Dobrovolskiene R Danyte E Verkauskiene R. Serum cystatin c as a biomarker for early diabetic kidney disease and dyslipidemia in young type 1 diabetes patients. Medicina (Kaunas) (2022) 58(2):218. doi: 10.3390/medicina58020218 35208542 PMC8878987

[25] Agrawal L Azad N Bahn GD Ge L Reaven PD Hayward RA. Long-term follow-up of intensive glycaemic control on renal outcomes in the veterans affairs diabetes trial (VADT). Diabetologia (2018) 61(2):295–9. doi: 10.1007/s00125-017-4473-2 PMC5747983 29101421

[26] Patney V Whaley-Connell A Bakris G. Hypertension management in diabetic kidney disease. Diabetes Spectr (2015) 28(3):175–80. doi: 10.2337/diaspect.28.3.175 PMC4536650 26300610

